# Increased Sensitization to Mold Allergens Measured by Intradermal Skin Testing following Hurricanes

**DOI:** 10.1155/2017/2793820

**Published:** 2017-04-09

**Authors:** Diego Saporta, David Hurst

**Affiliations:** ^1^Private Practice, Associates in ENT & Allergy, Elizabeth, NJ, USA; ^2^Tufts University, Boston, MA, USA

## Abstract

*Objective*. To report on changes in sensitivity to mold allergens determined by changes in intradermal skin testing reactivity, after exposure to two severe hurricanes.* Methods*. A random, retrospective allergy charts review divided into 2 groups of 100 patients each: Group A, patients tested between 2003 and 2010 prior to hurricanes, and Group B, patients tested in 2014 and 2015 following hurricanes. Reactivity to eighteen molds was determined by intradermal skin testing. Test results, age, and respiratory symptoms were recorded. Chi-square test determined reactivity/sensitivity differences between groups.* Results*. Posthurricane patients had 34.6 times more positive results (*p* < 0.0001) at weaker dilutions, all tested molds were found to be more reactive, and 95% had at least one positive test versus only 62% before the hurricanes (*p* < 0.0001); average mold reactivity was 55% versus 16% while 17% of patients reacted to the entire panel versus none before the hurricanes (*p* < 0.0001). The posthurricane population was younger (*p* < 0.001) and included more patients with asthma or lower respiratory symptoms (*p* < 0.05).* Conclusion*. Reactivity and sensitization to mold allergens increased compared to patients before the hurricanes. This supports climatologists' hypothesis that environmental changes resulting from hurricanes can be a health risk as reflected in increased allergic sensitivities and symptoms and has significant implications for physicians treating patients from affected areas.

## 1. Introduction

Northern New Jersey was affected by two catastrophic storms: Hurricane Irene in 2011 and Hurricane Sandy in 2012. The storms covered an extensive area leading to widespread destruction [1] with severe damage including loss of life [2]. Following these storms wide areas of the state remained underwater for several days.

Hurricanes are climactic events of catastrophic proportion. It has been clearly established that people exposed to mold after floods like the ones resulting from hurricanes frequently develop allergy symptoms and asthma [3–5]. Flooding following a hurricane is a key factor for mold growth and may be an important risk for exposed individuals [3].

Significant changes in intradermal skin testing (IDT) results during routine patient evaluation for allergic conditions were first noted beginning in late 2011 and more clearly during 2012. These changes were apparent for all tested allergens. A pattern of increased sensitivity and reactivity to molds was observed as tests yielded many more positive skin reactions and, frequently, a weaker allergen concentration was required to elicit skin responses. The study was conducted to determine if the change in reactivity and sensitivity observed in the tested patients was real. While demonstrating this point is relatively simple as will be shown in this paper, explaining why this is happening is more difficult. Certainly hurricanes Irene and Sandy produced the conditions that, as specifically mentioned in the literature [6–8], encourage mold growth. Could this potential mold growth have, in any way, affected the health of the exposed population? The fact is that, at the time of this writing, we find more complex patients whose health appears to be more affected. The intention of this paper is just to report about the observed changes in skin reactivity and sensitivity. This study is the first to compare the results of mold allergy testing performed on a population of a clearly defined geographical area before and after this area suffered the effects of two hurricanes.

## 2. Methods

Consecutive allergy charts were gathered and separated according to testing dates in two groups with one hundred cases each. Group A included those tested from 2003 to 2010, before the hurricanes, and Group B included those tested after the hurricanes in 2014 and 2015. Changes in skin reactivity had begun to be noted in late 2011 but appeared to be more evident each year following the hurricanes. To clarify the possible effect of this variable, we chose to eliminate all test results between the years 2011 to 2013 including only 2014 and 2015 for the posthurricane group, similar to the patient selection process used in our previous report on the effect of hurricanes on skin reactivity to dust and pollen [9]. Inclusion criteria were patients of any sex or age, with symptoms of allergic rhinitis with or without asthma, or lower respiratory symptoms (LRS) (i.e., symptoms pertaining to the lower airway but not formally diagnosed as asthma), provided that these patients were tested for the same panel of 18 molds.

The date of test, age, sex, presence of asthma or LRS, and test results were recorded. Any patients tested during both time periods were excluded to eliminate the potential effect of immunotherapy on skin reactivity of patients tested after a receiving immunotherapy. Test results were recorded as the diameter in millimeters (mm) of each test wheal at each dilution. A statistician compared results of the pre- and posthurricane testing. The chi-square test was used to determine the differences in reactivity/sensitivity between groups. A probability *p* < 0.05 was considered significant. Institutional Review Board (IRB) approval was obtained from Trinitas Medical Center, 225 Williamson Street, Elizabeth, NJ.

Intradermal Dilutional Skin Test (IDT) was performed on all patients according to guidelines from the American Academy of Otolaryngic Allergy (AAOA) [10, 11]. Briefly, successive serial 5-fold dilutions of each allergenic extract were prepared and conventionally labeled as Dilutions 1 to 6. Dilution 1 was five times weaker than the allergenic extract (1 : 5), Dilution 2 was 1 : 25, Dilution 3 was 1 : 125, Dilution 4 was 1 : 625, Dilution 5 was 1 : 3125, and Dilution 6 was 1 : 15,625.

The test consists of injecting 0.01 mL of allergen to produce a 4 mm wheal and measuring its diameter 10–15 minutes after injection. The first reactive wheal, called the End Point (EP), represents the minimal antigen concentration able to elicit a skin response 2 mm larger than the control which usually measures 5 mm. Mold allergens were obtained from the manufacturer as weight/volume (*W*/*V*) [12].

The mold panel consisted of the same 18 molds for both groups. The distribution of positive results in each dilution and the prevalence of individual tested molds were analyzed in Tables 1–6.

## 3. Results

Both groups had a similar distribution of males and females. Group B had more patients (18 or younger), 29% versus 12% in Group A (*p* < 0.01), and more patients with asthma or LRS, 39% versus 25% (*p* < 0.05) (Table 1). Group B had 3.5 times more total reactions (987 versus 285) (*p* < 0.0001) as well as more reactions to each of the dilutions tested. No positive test results occurred at Dilution 6 in Group A yet this occurred 5 times in Group B (Table 2).

Among both groups the majority of the positive reactions occurred for the dilutions with strong concentrations of allergen (Dilutions 1, 2, and 3): 743/987 (75%) for Group B and 277/285 (97%) for Group A (*p* < 0.0001). Twenty-five percent (244/987) of the positive reactions in Group B occurred with weak allergen concentration (Dilutions 4, 5, and 6), versus 3% in Group A (8/285) (*p* < 0.0001) (Table 3). This suggests that the skin sensitivity to molds in the posthurricane group had significantly increased.

Before the hurricanes, 62% of the patients had at least one positive mold test. After the hurricanes this increased to 95% (*p* < 0.0001). Before the hurricanes 38% of the patients were nonreactive to any mold compared to 5% of posthurricane patients (*p* < 0.0001). No patient reacted to all tested molds in Group A but 17% of the patients did so in Group B (*p* < 0.0001) and, among Group B, 44% of the patients reacted to 11 or more molds as compared to 4% in Group A (*p* < 0.0001) (Table 4). On average, 55% of molds were reactive in Group B as compared to 16% among Group A (*p* < 0.0001) (Table 5). In the posthurricane test example there are more positive reactions and the EPs tended to occur at weaker dilutions. Observation of this disparity is what motivated this study. Unlike our previous report on changes in skin reactivity following dust, epidermals, and pollen tests [9], mold tests rarely produced unusually large wheals.

The molds whose reactivity was greater than 1 standard deviation above average (15.83 + 6.6 for Group A and 55.0 + 8.0 for Group B) are marked in italic in Table 6. These included* Epidermophyton*,* Trichophyton, and Rhizopus* in Group A and the same three plus* Penicillium* in Group B.

## 4. Discussion

Hurricanes Irene and Sandy caused flooding that kept a wide geographical area underwater for several days [13, 14]. Buildings that remain wet for 48–72 hours following major hurricanes and floods frequently develop visible and extensive mold growth [4]. Some reports have been published following Hurricane Katrina [15, 16], but they did not evaluate the same geographical population both before and after that hurricane. In our case both groups of patients belong to the same geographical area, and both groups were tested by the same practitioner, using the same intradermal testing technique.

There is a strong association between exposure to water-damaged homes and development of upper and lower respiratory disease [3]. A worldwide increase in asthma and allergic rhinitis prevalence has been reported over the last two to three decades [17]. This increase appeared to be greater in children as asthma affects 20%–25% of the total population but 20%–40% of the childhood population [18]. Nasal allergy symptoms and asthma exacerbations are prominent manifestations of mold allergy [4]. Respiratory illness and asthma exacerbations have been noted following flooding [8] as a result of exposure to dampness or mold [4, 5, 7, 8], including reports of increased risk of respiratory arrest [8].

Our finding that posthurricane patients were reactive to many more allergens and at much weaker concentrations than in our preexposure group strongly suggests that the posthurricane population had become more sensitized and reactive to molds. The question that cannot be answered is as follows: Is this in any way related to exposure to a persistently damp environment? If so, similar changes might be expected to occur in other areas similarly affected by severe storms where flooding occurs.

The results showed an increased number of patients younger than 18 in Group B, as the percentage rose from 12% before the hurricanes to 29% after them (Table 1). Such change more likely reflects the results of their exposure and not necessarily selection bias, which would be in agreement with findings reported in the literature [18].

Group B had a higher incidence of positive results to both strong and weak mold allergen concentrations (Table 3). Positive responses at weak allergenic concentration were an infrequent finding before the hurricanes, occurring in only 3% of the cases as compared to 25% of the cases in Group B, an 8-fold increase. The difference in reactivity to allergy testing between the two groups in this study suggests that flooding after hurricanes may have been instrumental in sensitizing the exposed patient population to molds. Our finding that after the hurricanes 55% of patients reacted to molds versus 16% before and that the reactivity to each individual mold increased beyond that seen in Group A (Table 5) supports the hypothesis that the posthurricane population is more sensitized and reactive to molds which may be related to having been exposed to a persistently damp environment. This also supports the hypothesis that the posthurricane group represents a more sensitized and sicker population. Physicians working in other areas affected by flooding may find more patients with asthma or LRS, including a larger number of symptomatic children. Prevention strategies to minimize reactions during testing or treatment should be implemented. The limitations of a retrospective chart review are obvious in that all people from the same geographic would have been exposed to the hurricane, thus making it impossible to establish a local control group. We therefore chose populations representing pre- and posthurricanes. A prospective study, though ideal, is probably impossible, as predicting where a hurricane might occur is most difficult.

Our finding that posthurricane patients were reactive to many more allergens and at much weaker concentrations than in our preexposure group strongly suggests that the posthurricane population had become more sensitized and reactive to molds.

## 5. Conclusions

The results of 100 intradermal tests for molds on patients presenting to an allergy practice, after two major hurricanes affected the New Jersey coast, appeared to be different from the same tests performed on an identical number of patients before the hurricanes. The posthurricane test results suggest that these patients have experienced an increased reactivity and sensitivity to mold.

Overall, posthurricane patients presented at a significantly younger age (*p* < 0.01), had asthma or LRS more frequently (*p* < 0.05) (Table 1), and had a 3.5 times increase in the overall number of positive responses (987 versus 285) (*p* < 0.0001), including more positive reactions to weaker concentrations of allergen (Table 2). Sensitivity to 18 tested molds was markedly increased among posthurricane patients. Ninety-five percent in Group B versus 62% in Group A reacted to at least one mold (*p* < 0.0001) (Table 4). On average, 55% of molds were reactive in Group B as compared to only 16% among Group A (*p* < 0.0001) (Table 5). In Group B, 17% reacted to all tested molds, while no patient in the prehurricane group did so (Table 4).

It cannot be concluded that floods are directly responsible. Yet it is possible that floods following a major climatic event such as hurricanes may predispose patients to develop or worsen allergic rhinitis and/or lower respiratory airway disease. The increased mold sensitivity we report could be a confounding factor which might explain why more patients developed allergic rhinitis, asthma, or LRS following the hurricanes.

This study supports the hypothesis of climatologists that environmental changes resulting from hurricanes can be a significant health risk as suggested by the increased allergic sensitivities and symptoms among the exposed population which we document in this study.

These findings have significant implications for physicians who treat patients exposed to flooding disasters. Large scale studies are needed to confirm these observations.

## Figures and Tables

**Table 1:** Demographics.

	Male/female	Age (SD)	Age ≤ 18	LRS
A (2010)	40/60	44 (17)	12	25
B (2015)	36/64	34 (18)	29	39
*p* value	N/S	<0.001	<0.01	<0.05

Age (SD): average patient's age (standard deviation).

Age ≤ 18: number of patients, 18 years old or younger.

LRS: number of patients with asthma or lower respiratory symptoms.

A (2010): Group A, including tests performed in or before 2010.

B (2015): Group B, including tests performed in 2014 and 2015.

N/S: not significant.

**Table 2:** Number of positive skin tests per dilution.

Dilution	6 (1 : 15625)	5 (1 : 3125)	4 (1 : 625)	3 (1 : 125)	2 (1 : 25)	1 (1 : 5)	Totals
A (2010)	0	2	6	27	216	34	285
B (2015)	5	11	228	125	481	137	987^*∗∗*^

Numbers and percentages coincide as they are based on 100 cases.

Dilution 6 through 1 are 1 : 5 dilutions of the allergenic extract.

Totals: total number of positive mold tests in 100 tests (or 1800 individual mold tests).

A (2010): Group A, including tests performed in or before 2010.

B (2015): Group B, including tests performed in 2014 and 2015.

^*∗∗*^ *p* < 0.0001.

**Table 3:** Low reactors versus high reactors.

	Strong allergen concentration (Dil 1, 2, 3)	Weak allergen concentration (Dil 4, 5, 6)	Totals
A (2010)	277 (97%)	8 (3%)	285
B (2015)	743 (75%)^*∗∗*^	244 (25%)^*∗∗*^	987

Dil 1, 2, 3: first to third dilutions from the extract (1 : 5; 1 : 25; 1 : 125). These have a stronger allergen concentration.

Dil 4, 5, 6: fourth to sixth dilution form the extract (1 : 625; 1 : 3125; 1 : 15625). These have a weaker allergen concentration.

Totals: total number of positive mold tests in 100 tests (or 1800 individual mold tests).

A (2010): Group A, including tests performed in or before 2010.

B (2015): Group B, including tests performed in 2014 and 2015.

^*∗∗*^ *p* < 0.0001.

**Table 4:** Number of positive results per panel.

	Reactive tests	None	11 or more	18 (all reactive)
A (2010)	62/100	38	4	0
B (2015)	95/100	5	44	17
*p* value	*p* < 0.0001	*p* < 0.0001	*p* < 0.0001	*p* < 0.0001

Reactive tests: number of patients that had at least one positive individual mold test. It includes all the information in this table and patients that had between 1 and 10 positive results not shown in this table.

None: none of the 18 allergens reacted during the test.

11 or more: 11 or more of the allergens were found to be reactive during the test.

18 (all reactive): the 18 allergens reacted during the test (these are part of the “11 or more” column).

A (2010): Group A, including tests performed in or before 2010.

B (2015): Group B, including tests performed in 2014 and 2015.

All results are also percentages (100 patients in each group).

**Table 5:** Comparison of incidences of positive results for each individual mold allergen.

Molds	A (2010)	B (2015)
Alternaria	11	56
Aspergillus	15	45
Chaetomium	14	46
Cladosporium	9	49
Curvularia	10	44
Epicoccum	13	50
Fusarium	13	52
Smut	8	58
Helminthosporium	20	55
Mucor	19	51
Penicillium	15	64
Phoma	16	56
Pullularia	8	52
Rhizopus	33	65
Stemphylium	15	52
Candida	16	50
Trichophyton	25	68
Epidermophyton	25	72

*Totals*	285	987^*∗*^

*AVG (SD)*	15.83 (6.6)	55.00 (8.0)

AVG (SD): average number of times a mold was reactive in each group (standard deviation).

A (2010): Group A, including tests performed in or before 2010.

B (2015): Group B, including tests performed in 2014 and 2015.

All results are also percentages (100 patients in each group), so each number represents how many times that mold was reactive in 100 tests.

^*∗*^ *p* < 0.0001.

**Table 6:** Positive mold results arranged by incidence.

	% pos	A (2010)	B (2015)	% pos
(1)	*33*	*Rhizopus*	*Epidermophyton*	*72*
(2)	*25*	*Epidermophyton*	*Trichophyton*	*68*
(3)	*25*	*Trichophyton*	*Rhizopus*	*65*
(4)	20	Helminthosporium	*Penicillium*	*64*
(5)	19	Mucor	Smut	58
(6)	16	Candida	Alternaria	56
(7)	16	Phoma	Phoma	56
(8)	15	Aspergillus	Helminthosporium	55
(9)	15	Penicillium	Fusarium	52
(10)	15	Stemphylium	Pullularia	52
(11)	14	Chaetomium	Stemphylium	52
(12)	13	Epicoccum	Mucor	51
(13)	13	Fusarium	Candida	50
(14)	11	Alternaria	Epicoccum	50
(15)	10	Curvularia	Cladosporium	49
(16)	9	Cladosporium	Chaetomium	46
(17)	8	Pullularia	Aspergillus	45
(18)	8	Smut	Curvularia	44

% pos: number (and percentage) of times individual mold was reactive in each group.

A (2010): Group A, including tests performed in or before 2010.

B (2015): Group B, including tests performed in 2014 and 2015.

*Italic*: outliers (incidence higher than average + STD).
